# Cosmetic and Therapeutic Applications of Fish Oil’s Fatty Acids on the Skin

**DOI:** 10.3390/md16080256

**Published:** 2018-07-30

**Authors:** Tse-Hung Huang, Pei-Wen Wang, Shih-Chun Yang, Wei-Ling Chou, Jia-You Fang

**Affiliations:** 1 Department of Traditional Chinese Medicine, Chang Gung Memorial Hospital at Keelung, Keelung 20401, Taiwan; huangtsehung@gmail.com (T.-H.H.); chouweiling@gmail.com (W.-L.C.); 2 School of Traditional Chinese Medicine, Chang Gung University, Kweishan, Taoyuan 33303, Taiwan; 3 School of Nursing, National Taipei University of Nursing and Health Sciences, Taipei 11219, Taiwan; 4 Department of Medical Research, China Medical University Hospital, China Medical University, Taichung 40402, Taiwan; pan@mail.cgu.edu.tw; 5 Department of Cosmetic Science, Providence University, Taichung 43301, Taiwan; phageyang@gmail.com; 6 Pharmaceutics Laboratory, Graduate Institute of Natural Products, Chang Gung University, Kweishan, Taoyuan 33302, Taiwan; 7 Chinese Herbal Medicine Research Team, Healthy Aging Research Center, Chang Gung University, Kweishan, Taoyuan 33302, Taiwan; 8 Research Center for Food and Cosmetic Safety and Research Center for Chinese Herbal Medicine, Chang Gung University of Science and Technology, Kweishan, Taoyuan 33302, Taiwan; 9 Department of Anesthesiology, Chang Gung Memorial Hospital at Linkou, Kweishan, Taoyuan 33305, Taiwan

**Keywords:** fish oil, polyunsaturated fatty acid, omega-3, skin, cosmetology, dermatology

## Abstract

Fish oil has been broadly reported as a potential supplement to ameliorate the severity of some skin disorders such as photoaging, skin cancer, allergy, dermatitis, cutaneous wounds, and melanogenesis. There has been increasing interest in the relationship of fish oil with skin protection and homeostasis, especially with respect to the omega-3 polyunsaturated fatty acids (PUFAs), docosahexaenoic acid (DHA), and eicosapentaenoic acid (EPA). The other PUFAs, such as α-linolenic acid (ALA) and linoleic acid (LA), also show a beneficial effect on the skin. The major mechanisms of PUFAs for attenuating cutaneous inflammation are the competition with the inflammatory arachidonic acid and the inhibition of proinflammatory eicosanoid production. On the other hand, PUFAs in fish oil can be the regulators that affect the synthesis and activity of cytokines for promoting wound healing. A systemic review was conducted to demonstrate the association between fish oil supplementation and the benefits to the skin. The following describes the different cosmetic and therapeutic approaches using fatty acids derived from fish oil, especially ALA, LA, DHA, and EPA. This review summarizes the cutaneous application of fish oil and the related fatty acids in the cell-based, animal-based, and clinical models. The research data relating to fish oil treatment of skin disorders suggest a way forward for generating advances in cosmetic and dermatological uses.

## 1. Introduction

The effect of fish oils in disease prevention and management has been studied for more than 50 years. Fish oils, which are rich in fatty acids, show evidence of potential health benefits [1]. Large amounts of polyunsaturated fatty acids (PUFAs) are found in the extracts of fish oils. Docosahexaenoic acid (DHA) and eicosapentaenoic acid (EPA), which are long-chain omega-3 fatty acids, are the predominant PUFAs derived from fish oils. The interest in fish oils arose from the reports on Eskimos’ high dietary intake of fish oils associated with a very low occurrence of inflammation-related diseases and ischemic heart disorders [2]. Besides vitamins and minerals, fish oils are the most frequently used nutritional supplements in older adults >65 years of age [3]. The PUFAs in fish oils have proved to be beneficial for treating rheumatoid arthritis, psoriasis, ulcerative colitis, asthma, Parkinson’s disease, osteoporosis, diabetes mellitus, cardiovascular events, cancers, and depression [4]. PUFAs also demonstrate beneficial activity on the development of the nervous, immune, visual, and cutaneous systems in infants [5].

It is believed that the bioactivities of fish oils are chiefly due to the effect of PUFAs. The epidemiological studies show a significant improvement of asthma symptoms in patients receiving fish oil supplements rich in DHA and EPA [6]. The use of omega-3 fatty acids in fish oil capsules has been associated with a reduction in plasma triglyceride concentration, leading to the decreased incidence of hyperlipidemia [7]. The PUFA supplementation can diminish the risks of cardiovascular diseases such as thrombosis, high blood pressure, and low high-density-lipoprotein cholesterol [8]. The meta-analysis studies have shown that fish oil consumption and dietary omega-3 PUFAs decrease the risk factor of type 2 diabetes mellitus via enhanced insulin sensitivity [9]. The consumption of fish oil containing a high level of PUFAs can play a role in cancer prevention and therapy [10]. The anticancer effect of omega-3 PUFAs is ascribed to the capability of downregulating proinflammatory eicosanoid synthesis from cyclooxygenase-2 (COX-2) [11]. The PUFAs from fish oils or cod liver oils can also be employed with a high level of safety as natural antibacterial and anti-infectious agents [12]. Intravenous lipid emulsions are a component of parenteral nutrition used as a resource for essential fatty acids for supplying energy to patients. Soybean oil is the traditional source of lipid emulsions. However, a high percentage of omega-6 PUFAs in soybean oil contributes to the immunosuppressive effect [13]. Recently, fish oil has been used to replace soybean oil in lipid emulsions to reduce the possible risk of inflammatory complications [14].

The benefits of fish oil are primarily attributed to omega-3 fatty acids, found mainly in oily fish. Since fish liver is high in lipids, most fish oils are derived from the hepatic region. The fish oil formulations are available from different species, including shark, tuna, lemuru, capelin, polar cod, saithe, mackerel, herring, and sprat [15]. The composition of omega-3 PUFAs in the commercially available products depends upon the source of the fish, the body part of the fish, and the extraction methods employed. The public awareness of the need to take fish oils to benefit the skin has been identified with the increasing research in the fields of cosmetology and dermatology. The lack of PUFAs can cause increased transepidermal water loss (TEWL), resulting in skin barrier function deficiency [16]. PUFA insufficiency also elicits the upregulation of proliferative keratins (K6 and K16) and inflammation-related keratin (K17) [17]. These findings highlight the importance of PUFAs for epidermal homeostasis. Some topically applied formulations of fish oil extract are developed for cosmetic and pharmaceutical products [18]. The application of fish oil is expected to change the landscape of dermatological therapy. In this review, we highlight recent advances in the application of fatty acids in fish oils for preventing or treating skin-associated disorders. Fish oil-based therapy is reported to treat various diseases such as photoaging, skin cancers, dermatitis, melanogenesis, and cutaneous infection. The promising perspective in this emerging application is also discussed.

## 2. Fatty Acids of Fish Oil

**Figure 1:**
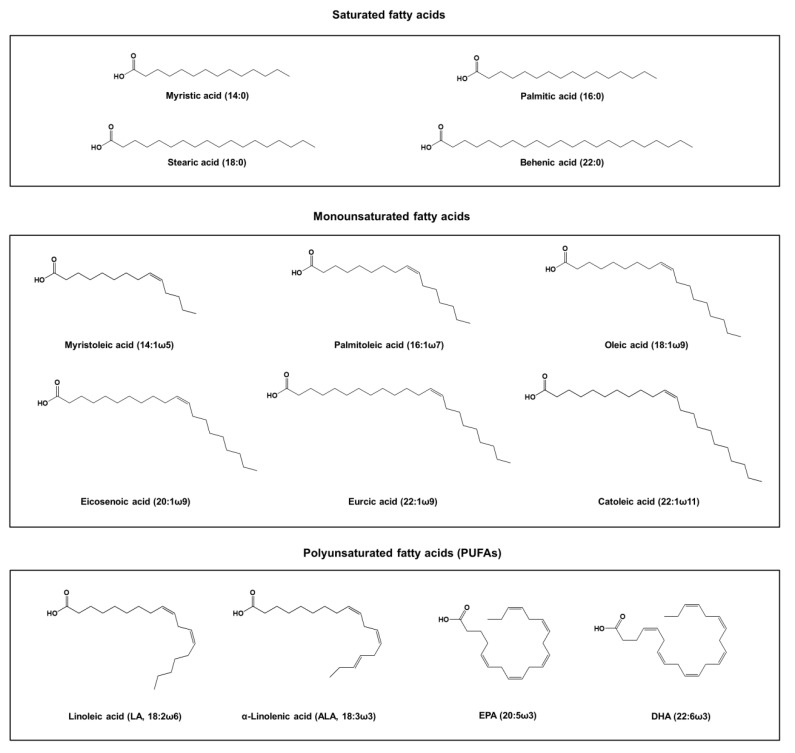
The chemical structures of fatty acids derived from fish oil.

Fish oil is abundant in fats and fatty acids, which can contain vitamin A, vitamin D, cholesterol, monoglycerides, diglycerides, triglycerides, free fatty acids, phospholipids, and sterylesters [19,20]. Among these components, esters are the main ingredient and they gain most attention for being associated with the bioactivities. Fatty acids in fish oil are present in both the neutral lipid and free acid forms. The typical fatty acid composition of the fish oil extract can be divided into saturated fatty acids, monounsaturated fatty acids, and PUFAs. The main saturated fatty acids found in fish oil include myristic acid (14:0), palmitic acid (16:0), stearic acid (18:0), and behenic acid (22:0) [21]. The monounsaturated fatty acids in fish oil include myristoleic acid (14:1ω5), palmitoleic acid (16:1ω7), oleic acid (18:1ω9), eicosenoic acid (20:1ω9), gadoleic acid (20:1ω11), erucic acid (22:1ω9), and catoleic acid (22:1ω11). The major PUFAs in fish oil are presented as linoleic acid (LA, 18:2ω6), α-linolenic acid (ALA, 18:3ω3), DHA (22:6ω3), and EPA (22:5ω3). Figure 1 depicts the chemical structures of these fatty acids. The PUFA content in marine fish is greater than that in freshwater fish [22]. The marine fish oil usually contains a considerable number of PUFAs with the longer chains (>20 carbon atoms), whereas the freshwater fish oil reveals the most PUFAs with fewer chain lengths (<20 carbon atoms) [23].

Both omega-6 and omega-3 PUFAs are essential fatty acids because the mammalian cells lack the desaturase enzymes capable of placing double bonds at the positions of ω6 and ω3 [24]. Both PUFAs should be obtained from diet and supplementation. The omega-6 and omega-3 fatty acids are needed for normal growth and health maintenance. They are metabolized via the lipoxygenase and cyclooxygenase (COX) pathways. The various types of metabolites are essential in the regulation of inflammatory and immune responses. LA and ALA, the PUFAs with the shorter chain length (18 carbon atoms), are the precursors to the biosynthesis of the omega-6 and omega-3 PUFAs with the longer chain lengths, respectively [25]. Both fatty acids are abundant in fish oil, peanuts, canola oil, and vegetable oil [26]. LA and ALA have similar chemical structures but different functions in the human body.

LA is a dominant omega-6 PUFA in fish oil. This fatty acid is essential for growth, reproduction, and skin function. It can be metabolized to γ-linolenic acid (GLA), dihomo-GLA, prostaglandin (PG)E1, and arachidonic acid (AA). The eicosanoids, such as PGE2 and leukotriene B4, are derived from AA. These eicosanoids are involved in the inflammation and allergic response in cutaneous tissue [27,28]. LA is the richest fatty acid in the epidermal layer. It is also the precursor to ceramide synthesis [29]. Ceramide is a predominant material of the intercellular stratum corneum lipid matrix producing the skin’s permeability barrier. Several omega-3 fatty acids are produced from ALA. These include DHA, EPA, and docosapentaenoic acid [30]. ALA is largely found in vegetable oils, zooplankton, phytoplankton, and fish oils. ALA is fundamental to visual and brain functions through its effect on membrane fluidity because PUFAs and their derivatives are principally located in the cell membrane phospholipids [31]. The derivatives of ALA can modify the immune response of the epidermis via affecting the T cells, acting on Toll-like receptors, and stimulating caspase cascades that relieve inflammatory dermatoses such as acne, psoriasis, dermatitis, lupus, and skin cancers [32]. The mechanisms of ALA and the derivatives for inflammation inhibition are based on barrier function maintenance, stratum corneum maturation, stratum corneum differentiation, proinflammatory eicosanoid inhibition, lamellar body formation, lipoxygenase inhibition, and cytokine suppression [33].

**Figure 2:**
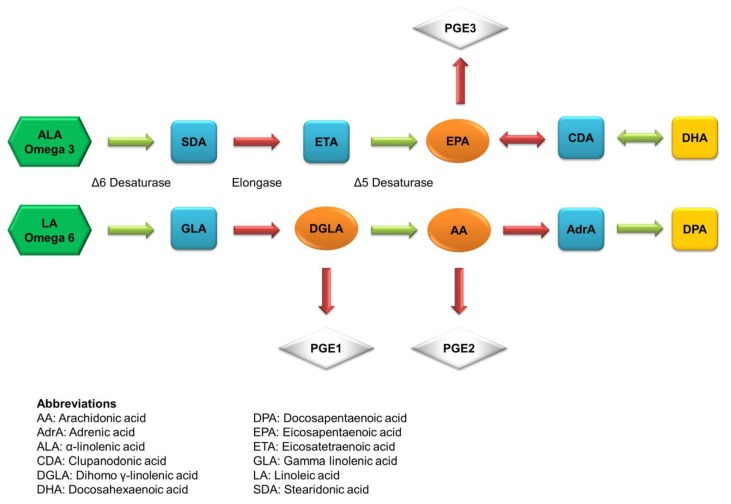
The possible metabolism pathways of essential fatty acids in the body.

The main derivatives of the ALA metabolism are DHA and EPA. Figure 2 illustrates the metabolic pathways of ALA to produce DHA and EPA. They always act as the indicator components of fish oil. The first double bond of both long-chain PUFAs is located at the third carbon atom from the methyl end. DHA and EPA have been used nutritionally and therapeutically in several diseases with variable success. DHA mainly resides in the retina and brain to maintain the membrane order and the activity of membrane-bound enzymes. DHA deficiency occurs during aging and dementia, impairs memory and learning, and promotes age-related neurodegenerative diseases, including Alzheimer’s disease. DHA is reported to have the ability of tumor inhibition and chemoprevention against colon cancer, prostate cancer, pancreatic cancer, and breast cancer [34]. EPA can compete with AA, an omega-6 PUFA, through the same metabolic pathways, but it produces eicosanoids that are functionally different from the AA derivatives. EPA is less potent as an inflammatory mediator compared to AA. Because of the competitive relationship, EPA can restrain AA-derived PGE2 synthesis [35]. The same as DHA, EPA is used to prevent or treat neurodegenerative diseases because of the anti-inflammatory and neuroprotective activity [36].

## 3. Fatty Acids for Skin Disorder Prevention and Treatment

Recent application of fatty acids found in fish oil in skin-related diseases includes therapies for photoaging, cancer, dermatitis, wound healing, and melanogenesis. The use of PUFAs ameliorates the symptoms of the skin diseases. Some fatty acids have been approved for clinical use or are under clinical trial for preventive or therapeutic use. In addition, some fish-oil-containing formulations are approved to manage various skin diseases in cell-based and animal studies. The following describes the different cosmetic and therapeutic approaches of fatty acids derived from fish oil, especially LA, ALA, DHA, and EPA. The pharmacodynamic outcomes of the fatty acids are the main evaluation platforms used to define the preventive or therapeutic effect for our description.

### 3.1. Photoaging

**Figure 3:**
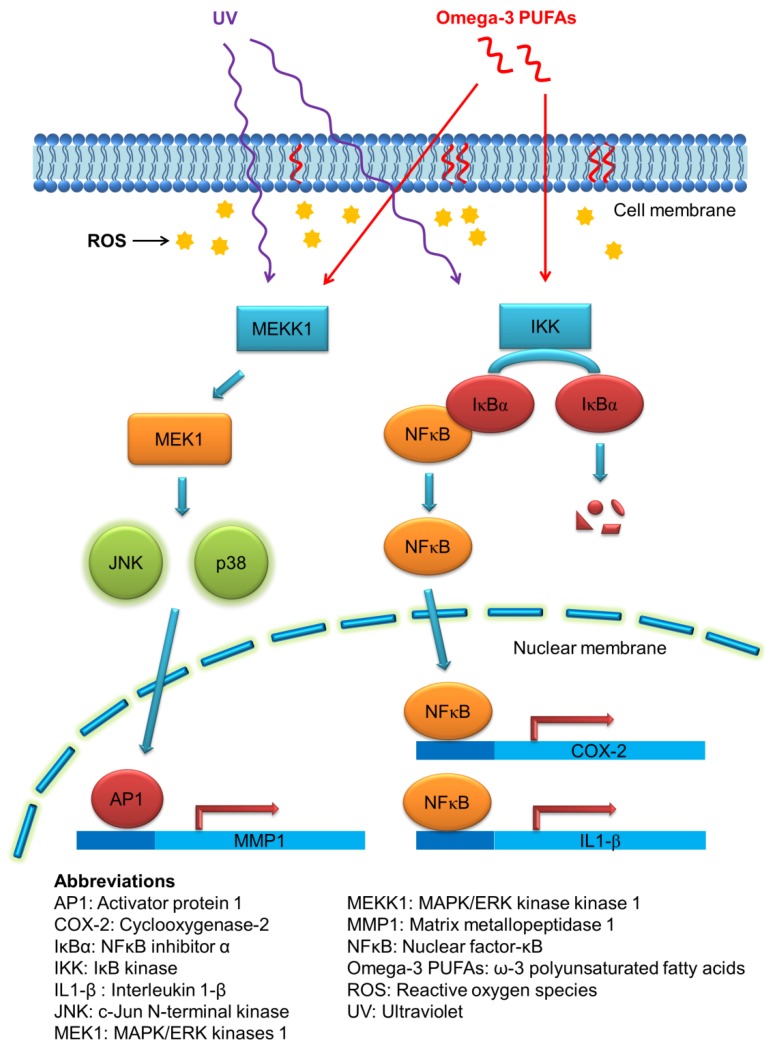
The possible mechanisms of the photoprotective capability of omega-3 PUFAs.

Cutaneous aging can be divided into chronological aging and photoaging. Photoaging is activated via the human skin damage attributable to repeated ultraviolet (UV) exposure from sunlight. UV irradiation elicits both acute and chronic adverse effects on the skin. These include sunburn, photosensitivity, inflammation, immunosuppression, and photocarcinogenesis [37]. UV exposure of the skin creates reactive oxygen species (ROS), leading to the massive infiltration of immune cells such as neutrophils and macrophages in viable skin [38]. One of the key proteins mediating the inflammatory signals in UV-induced injury is cyclooxygenase-2 (COX-2), which catalyzes the biosynthesis process of prostaglandins [39]. In addition to sunscreens, some photoprotective agents are needed to provide advantages against UV-induced skin damage. The fatty acids derived from fish oil have been considered to be associated with the skin’s photoprotection. Omega-3 PUFAs can decrease the production of proinflammatory eicosanoids through direct competition with the metabolism of AA [40]. The other mechanisms of omega-3 PUFAs for suppressing UV-induced keratinocyte damage can be the regulation of COX-2, NF-Κb, and mitogen-activated protein kinase (MAPK)/extracellular-signal-regulated kinase (ERK) pathways [41]. Figure 3 illustrates the possible mechanisms of the photoprotective capability of PUFAs.

Interleukin (IL)-8, a proinflammatory cytokine belonging to the C-X-C chemokine subfamily, is of major significance in the mediation of UVB-induced keratinocyte inflammation [42]. Storey et al. [43] investigated whether the inhibition of UVB-induced inflammation by DHA and EPA is mediated by the modulation of IL-8 in keratinocytes and skin fibroblasts. In keratinocytes, DHA and EPA reduced the IL-8 level by 65% and 66% after UVB irradiation at 100 mJ/cm^2^. A similar pattern was observed in fibroblasts. Oleic acid showed no influence on IL-8 release. Serini et al. [44] explored the ability of DHA to influence the resistance to UV-activated apoptosis in keratinocytes. DHA reverted HaCaT cell resistance to UV-induced apoptosis, increasing the Bax/Bcl-2 ratio and caspase-3 activity, and decreased COX-2 by the inhibition of human antigen R (HuR), a COX-2 mRNA stabilizer in keratinocytes. The incorporation of DHA at 50 μM to the UV-irradiated cells decreased cytoplasmic HuR by 71%. UV-induced metalloproteinases (MMPs) elicit connective tissue damage, resulting in the skin’s aging and wrinkling [45]. Kim et al. [46] investigated the effect of EPA on UV-induced MMP-1 expression in dermal fibroblasts. The broadband UV (275–380 nm) at 25–75 mJ/cm^2^ was used as the UV source. Pretreatment of EPA at 5 and 10 μM inhibited MMP-1 by 33% and 79% compared to the UV-treated group, respectively. EPA could suppress MMP-1 by inhibiting the ERK and Jun-N-terminal kinase (JNK) pathways. On the other hand, AA and oleic acid pretreatment slightly increased or did not affect MMP-1 expression.

The photoaging animal models were developed to examine the impact of omega-3 fatty acids on cutaneous photoprotection. The UVB irradiation at 500 mJ/cm^2^ for 20 min was employed to test the inhibitory activity of EPA on mouse ear edema [47]. The daily oral dose of 300 mg/kg EPA for 2 weeks could suppress the ear edema. However, no function was observed in the groups receiving ≈30–100 mg/kg EPA. No amelioration of ear edema was detected with the use of oral DHA and safflower oil in this case. Topical administration provides a direct and efficient way to deliver the active agents into the cutaneous nidus with higher bioavailability than the oral route. Rahman et al. [48] investigated the inhibitory effect of topically applied DHA on UVB-induced skin inflammation in hairless mice. Topical pretreatment of DHA (2.5 and 10 μmol) significantly decreased COX-2 and nicotinamide adenine dinucleotide phosphate (NADPH): oxidase-4 (NOX-4) in mouse skin. Both COX-2 and NOX-4 are important in evoking oxidative stress and inciting inflammation. The molecular mechanisms of this inhibition could be the suppression of UVB-induced NF-κB activation and COX-2/NOX-4 expression by blocking the phosphorylation of stress-activated kinase-1 (MSK1), which is a kinase downstream of ERK and p38. The transcription factor Nrf2 is a major regulator of anti-inflammatory and antioxidant gene expression [49]. UVB exposure (180 mJ/cm^2^) for 23 weeks was used to enhance COX-2 and Nrf2 expression in hairless mouse skin to determine the effect of topical DHA against photoaging [50]. Topical DHA application (10 μmol) before irradiation induced the expression of Nrf2 target protein heme oxygenase-1 (HO-1) in the skin and protected against UVB-activated inflammation and papillomagenesis.

The anti-photoaging effect of PUFAs occurs in cell-based and animal-based studies as well as in human studies. In the early 1990s, a short-term supplementation of fish oil was conducted in humans [51]. The volunteers received oral fish oil containing rich omega-3 PUFAs (1.2 g DHA and 2.8 g EPA) each day for 4 weeks. At the end of the experiment, there was a significant increase in the minimal erythema dose (MED) to UVB with a decreased serum triglyceride level to 40 mg/dL. Rhodes et al. [52] examined the photoprotective effect of fish oil on light-sensitive patients. Thirteen patients with polymorphic light eruption received oral supplementation of fish oil containing DHA, EPA, palmitic acid, palmitoleic acid, and oleic acid for 3 months. The mean MED increased from 19.8 to 33.8 mJ/cm^2^ by dietary fish oil. PGE2 increased from 8.6 ng/mL in the sham group to 27.2 ng/mL after UVB treatment. The PGE2 level decreased to 4.1 and 9.6 ng/mL in the control and irradiated skin, respectively. Puglia et al. [53] evaluated the percutaneous absorption and the photoprotective effect of three fish oils rich in DHA and EPA, including mackerel, sardine, and horse mackerel. The in vitro skin permeation showed that the fish oil from sardines facilely penetrated into the skin as compared to the oil from the others. The clinical experiment was carried out in ten volunteers with the irradiation of UVB at doubled MED. The topical application of combined sardine extract and ketoprofen, an anti-inflammatory drug, inhibited the UVB-induced erythema by 60.5%, which was greater than the inhibition achieved by sardine oil extract (24.5%) and ketoprofen (46.6%) alone.

The influence of dietary EPA on UVB-generated PGE2 and proinflammatory cytokines was examined in a double-blind, randomized study [54]. Twenty-eight volunteers received 4 g daily of 95% ethyl esters of EPA or oleic acid for 3 months. The group of EPA but not oleic acid exhibited a significant enhancement of MED. PGE2’s increase by UVB (26.5 pg/mL) could be eliminated by EPA (19.3 pg/mL), approximating the baseline data (14.0 pg/mL). However, this study demonstrates no evidence that the reduced sunburn response by EPA was mediated by cytokines. The PUFAs are not always beneficial to various facets of cutaneous photoaging. Langerhans cells are sentinels of the immune system in the skin. Following UV exposure, the loss of this cell in the epidermis is detected [55]. In a double-blind, randomized controlled study of 79 females, Pilkington et al. [56] explored the effect of dietary EPA on epidermal Langerhans cells and prostaglandin D2 (PGD2). The healthy volunteers received EPA-rich capsules (5 g EPA) or control lipid (glyceryl tricoprylate caprate) for 12 weeks. The clinical data revealed that there was no impact of EPA supplementation on the Langerhans cell number and the PGD2 level after UV irradiation compared to the control. There was no evidence that EPA reduced UV suppression on skin immunity through this mechanism. Kim et al. [57] investigated whether topical EPA could inhibit both UV-induced photoaging and intrinsic aging to young and aged volunteers, respectively. The buttock skin was irradiated with UVB at doubled MED (about 70–90 mJ/cm^2^). UVB increased epidermal thickness by 214%, and topical EPA reduced the thickness by 72%. UVB light reduced the procollagen expression to 18% of the untreated control. EPA could restore the procollagen level to 46% of the control group. This PUFA also attenuated COX-2, MMP-1, and MMP-9 elevated by UVB.

In addition to the PUFAs with the longer chains (>20 carbon atoms), the short-chain PUFAs are useful in suppressing UV-induced cutaneous injury. The effect of orally and topically applied oils enriched with LA and ALA on UV-induced damage was compared in hairless mice [58]. Both LA and ALA lowered the erythema score compared to the basal cream after topical administration. On the other hand, dietary ALA demonstrated greater erythema inhibition than LA by the oral route. The PGE2 expression increased 8-fold after UVB exposure. Dietary LA did not diminish the increased PGE2, whereas the PGE2 level in the ALA group was 75% lower than that in the LA group. The results indicated that both omega-6 and omega-3 PUFAs could play a role in the constraint of UVB-elicited lesions. Conjugated LA represents the positional and geometrical isomers of LA. These isomers are reported to block LA metabolism to γ-linolenic acid in omega-6 PUFAs [59]. Conjugated LA is beneficial for decreasing white adipose tissue weight in subcutaneous tissue, an implication of obesity management [60]. Storey et al. [61] examined the capability of conjugated LA to inhibit IL-8 and PGE2 in UV-irradiated keratinocytes. Supplementation of keratinocytes with *c*9,*t*11-conjugated LA downregulated UVB-induced IL-8 from 37.11 to 14.17 ng/mg. Another LA isomer, *tt*-conjugated LA, reduced UVB-induced PGE2 release from 4.8 to 1.6 pg/mg. According to the above description, it is believed that oral or topical application of PUFAs from fish oil is helpful in preventing or treating skin aging. This is the appeal of many cosmetic products. These fatty acids are included in some skin creams for cosmetic purposes [62]. The detailed information about the fatty acids existed in fish oils for attenuating cutaneous photoaging is summarized in Table 1.

### 3.2. Cutaneous Carcinogenesis

Skin cancers are generally classified into melanoma and non-melanoma skin carcinoma (NMSC). UVB radiation is the most prevalent risk factor responsible for the development of skin cancers. However, it has been recognized that UVA is also responsible for procarcinogenic action on the skin [63]. The oxidative stress and continuous inflammation are responsible for the main pathologic generation in UV-induced skin photocarcinogenesis [64]. Another important contribution of UV to developing skin cancers is the suppression of cutaneous immunity [65]. The PUFAs from fish oil are found to inhibit both the initiation and promotion phases of cutaneous carcinogenesis. Both DHA and EPA were tested for their effectiveness on premalignant keratinocyte apoptosis [66]. The HaCaT cell growth was significantly inhibited by both omega-3 fatty acids at 30 and 50 μM. DHA or EPA at 50 μM lowered the number of viable keratinocytes by 60–80% compared to the control. The combined anti-cancer drugs and dietary PUFAs may be advantageous to achieving synergistic inhibition on carcinogenesis. Chiu et al. [67] elucidated the effect of non-steroidal anti-inflammatory drugs (NSAIDs) and DHA combination for melanoma cell growth inhibition. Celecoxib and indomethacin revealed additive effects on DHA-induced inhibition. Aspirin promoted DHA-induced growth inhibition by 43% at 480 μM. The IC_50_ of DHA on melanoma growth inhibition was 160 μM. Piroxicam could decrease the IC_50_ to 40 μM. The administration of high-dose COX inhibitors would create the unwanted adverse effects [68]. An ideal strategy to attenuate the risk raised by NSAIDs is the use at low dose with the supplement of chemopreventive agents such as long-chain PUFAs.

Imiquimod is a toll-like receptor 7/8 agonist prescribed as a topical drug for treating actinic keratosis, skin warts, and malignancy [69]. Nevertheless, it is known to cause severe skin inflammation. Based on the concept of synergistic carcinoma inhibition for lowering the administered dose, fish oil was used in combination with imiquimod to treat human basal (BCC) and squamous carcinoma cells (SCC) [70]. The fish oil utilized in this case was composed of 21% DHA and 42% EPA. The combined imiquimod and fish oil demonstrated greater cell viability inhibition and immunomodulatory potency as compared to imiquimod alone. The pure DHA or EPA was more potent than fish oil for the immunomodulatory effect against the carcinoma cells. The omega-3 PUFAs served as the inducers of IL-10, an anti-inflammatory cytokine, and as the suppressors of IL-6 and TNF-α to depress cell growth. Rehman and Zulfakar [71] further developed the imiquimod-loaded fish oil bigel colloidal delivery system for treating skin cancer in a mouse model. Bigel is defined as an intimate hydrogel/oleogel colloidal semisolid vehicle for topical application [72]. Fish oil as a source of DHA and EPA is also employed as the permeation enhancer to improve drug delivery into the skin [73]. After topical delivery of the imiquimod formulations on the mouse bearing the skin tumor induced by 7,12-dimethylbenz(a)anthracene (DMBA), there was a significant reduction of the tumor size by bigel (2.07 mm) and the commercial imiquimod cream (1.98 mm) as compared with the sham control (6.48 mm). The mouse treated with bigel exhibited greater IL-10 expression (40.86 pg/mL) than commercial cream (27.82 pg/mL) and the control (0.63 pg/mL). The fish oil rich in omega-3 PUFAs was topically applied on the mouse skin with papilloma prompted by benzo(a)pyrene and croton oil [74]. Fish oil blocked the binding of benzo(a)pyrene to DNA, resulting in the reduction of the mean papilloma number per mouse from 6.0 to 3.1. In addition to topical delivery, the oral administration of a high-fat diet containing fish oil rich in omega-3 PUFAs in mice also repressed UVB-induced carcinogenesis [75]. Fish oil intake could increase the latency to the development of UVB-induced tumor and decrease the size of the papilloma, keratoacanthoma, and carcinoma in mice by 98%, 80%, and 83%, respectively. The tumor inhibition was not observed in the group receiving the high-fat diet rich in omega-6 fatty acids.

The varied effects of different classes of dietary fatty acids on cutaneous carcinogenesis suggest that fatty acid composition is an important determining factor in tumor development. In the previous study [76], the association between dietary n-3 and n-6 fatty acid intake and the risk of SCC was explored. The results taken from a population-based case-control study demonstrated a consistent tendency toward a lower SCC risk with higher omega-3 PUFA consumption. The risk of SCC decreased following the increase of omega-3/omega-6 ratio fatty acid intake. In another case-control study of melanoma patients [77], the higher uptake of fish oil rich in omega-3 fatty acids was defined as more than one portion a week and was associated with a lower risk of melanoma development. This result was based on the participants’ completion of a food frequency questionnaire. A phase 2 open-label clinical study was performed to investigate the response rate and safety of DHA-paclitaxel conjugate in metastatic melanoma patients [78]. Paclitaxel is an anti-melanoma drug with a narrow therapeutic window. DHA-paclitaxel is a covalent conjugate showing a greater therapeutic index than paclitaxel alone [79]. This conjugate had been successfully targeted to the tumor with minimal deposition in normal tissue [80]. Thirty patients were enrolled to receive a DHA-paclitaxel intravenous infusion at 500 mg/m^2^/week for 5 weeks. The median survival period could be prolonged to 14.8 months. It is indicated that the weekly DHA-paclitaxel is a solidly tolerable single agent for melanoma patients.

Conjugated LA was orally administered to the mouse bearing skin cancer to determine the presence of peroxisome proliferator-activated receptor (PPAR)-δ and keratinocyte fatty acid binding protein (K-FABP), which are involved in cutaneous tumor promotion [81]. The skin malignancy was developed by topical administration of DMBA and 12-O-tetradecanoylphorbol-13-acetate (TPA). The results showed that PPAR-δ and K-FABP in the mRNA level were decreased by feeding the diet containing 0.5–1.5% conjugated LA. It is suggested that conjugated LA inhibited skin tumor promotion via the mechanism of PPAR-δ. Table 2 depicts the related information of fatty acids existed in fish oils for preventing or treating cutaneous carcinogenesis.

### 3.3. Dermatitis

Dermatitis is an inflammatory and itchy skin condition with a predilection for cutaneous flexure. It is characterized by symptoms such as intense pruritus, erythematous papules with excoriation, vesicles over erythematous skin, thickened plaques of skin, accentuated skin marking (lichenification), and fibrotic papules (prurigo nodularis) [82]. The symptoms of dermatitis can cause barrier function defects, followed by the invasion of bacteria and allergens, as well as transepidermal water loss and fat loss. After the diagnosis based on developed criteria, dermatitis can be classified according to several types: atopic dermatitis, allergic contact dermatitis, irritant contact dermatitis, seborrheic dermatitis, discoid eczema, and frictional lichenoid dermatitis [83]. Fish oil and the related fatty acids are reported to be useful for ameliorating dermatitis symptoms. Barcelos et al. [84] demonstrated the reduction of cutaneous dryness and pruritus by oral supplementation of fish oil in rats. Dry skin is a consequence of the subtraction of epidermal water content due to stratum corneum barrier function loss [85]. A 30% increase in cutaneous hydration was detected after fish oil consumption for 60 days, persisting at 90 days in the acetone-induced dry skin animal model. The itch-related scratching behavior was also eliminated after supplementation. The 90-day supplementation led to an increased uptake of DHA (1.8×), EPA (2.2×), and docosapentaenoic acid (1.7×) into the skin.

Trimellitic anhydride is broadly used in the plastics industry but can prompt cutaneous allergy via immune cell accumulation such as in atopic dermatitis [86]. In order to ameliorate the cutaneous allergy sensitized by trimellitic anhydride in rats, omega-3 PUFAs (600 mg/kg) was orally administered [87]. The results displayed a significant reduction in the ear thickness, cutaneous eosinophils, and mast cells after fatty acid administration. Fatty acids also decreased the inducible nitric oxide synthase (iNOS) expression and collagen fibers. Weise et al. [88] investigated the amelioration of dietary DHA and AA with respect to the severity of ovalbumin-induced dermatitis in mice. The mice consumed a daily dose of 24 mg/kg DHA and/or 48 mg/kg AA. The clinical outcome of dermatitis was significantly reduced by combined DHA and AA. The improvement was accompanied by a significant decrease in Ki67 expression to 62.5% of the control. The elevated IL-10 was also found in the cutaneous lesion of the DHA/AA-treated animal.

The 18:3 PUFAs, especially GLA, can be the dietary supplementation to improve dry skin and dermatitis. PGE1 and 15-hydroxyeicosatrienoic acid converted from GLA via dihomo-GLA possess anti-inflammatory characteristics. GLA supplementation was investigated to reverse epidermal hyperproliferation [89]. The consumption of GLA-rich borage oil modified fatty acid metabolism and increased the skin barrier function [90]. In the previous report [91], GLA-rich oil was incorporated into the food for oral consumption in 130 subjects with mild atopic dermatitis. After 4 weeks, the GLA group revealed lower TEWL and a higher stratum corneum index compared to the control. No significant side effects were found after GLA administration. The mechanism of skin barrier recovery has been associated with the possible generation of anti-inflammatory metabolites from GLA. The dihomo-GLA concentration in the serum of atopic dermatitis patients was lower than that of the healthy control [92]. Dihomo-GLA is one of the active metabolites of GLA. Since GLA is sometimes not effectively converted into dihomo-GLA in dermatitis patients, Kawashima et al. [93] examined whether oral delivery of dihomo-GLA prevented dermatitis-like lesions in NC/Nga mice. The clinical severity score and scratching behavior manifested lower levels in the mice fed dihomo-GLA. The total plasma immunoglobulin (IgE) was significantly lower in the dihomo-GLA group (15.6 μg/mL) than in the control (64.2 μg/mL). In another study utilizing NC/Tnd mice as the animal model [94], dietary dihomo-GLA but not AA and EPA suppressed the development of dermatitis-like lesions. The application of dihomo-GLA upregulated prostaglandin D1 (PGD1), resulting in the subsequent suppression of IgE-mediated degranulation. The amount and duration of scratching were lessened by dihomo-GLA supplementation. The description of the fatty acids existed in fish oils for preventing or treating dermatitis is shown in Table 3.

### 3.4. Cutaneous Wounds

Skin wounds, such as second-degree burns, chronic wounds, and ulcers, have affected millions of people worldwide. Though there are several skin replacement products and wound dressings for promoting wound healing, the development of efficient and safe approaches for cutaneous wound healing is urgently needed [95]. Wound healing is divided into three stages: inflammatory response, proliferation, and maturation [96]. The cellular and molecular processes in the inflammatory phase of wound healing are initiated and amplified to a large degree by proinflammatory cytokines. The synthesis and activity of cytokines can be regulated by PUFAs [97]. These fatty acids have been proved to play a key role in cell membrane structure and anabolic events during skin tissue reconstruction. It is possible that omega-3 and omega-6 PUFAs modulate or enhance local inflammatory response at wound areas, accelerating the healing rate [98]. Shingel et al. [99] described the preparation of a solid emulsion gel for cell-targeted PUFA delivery to skin wounds. The emulsion hydrogel is a combination of a protein-stabilized lipid emulsion and a hydrogel vehicle. The full-thickness skin wound reaching muscular fascia was created in the pig with a 25-mm diameter. The wound treated with the fish-oil-containing gel showed a faster wound closure compared to the gel containing olive oil. A significant wound closure was achieved at day 2 and day 10 by fish oil and olive oil, respectively. Fish oil was found to stimulate early angiogenesis for promoting wound healing.

SMOFlipid, which acts as parenteral nutrition, is a lipid emulsion mixture with four lipid resources: medium-chain triglycerides, soybean oil, olive oil, and fish oil [100]. Peng et al. [101] assessed the efficacy of SMOFlipid rich in omega-3 PUFAs on wound healing in rats. SMOFlipid was intravenously injected at 0.2 mL/kg immediately after excision for 72 h. The SMOFlipid accelerated the healing process more than the placebo by reducing the surface area of the wound by ≈20–25% at day 3. The IL-10 level and collagen fiber organization were greater in the SMOFlipid group than the placebo after 48 h of treatment. The topical use of DHA (30 μM) hastened the skin wound healing through the inflammatory activity modulation in rats [102]. The wound in the DHA group was completely healed at day 15, whereas a 30% wound was still unhealed in the control. Upon DHA treatment, the wound healing was accompanied by the activation of the G-protein-coupled receptor (GPR)120, a receptor for DHA with anti-inflammatory activity. The expression of transforming growth factor β (TGF-β) and keratinocyte marker involucrin was upregulated after DHA application. The DHA analogues 14*R*,21-dihydroxy-DHA and 14*S*,21-dihydroxy-DHA were obtained from DHA catalysis by 12-lipoxygenase and cytochrome P450. 14*R*,21-dihydroxy-DHA and 14*S*,21-dihydroxy-DHA significantly increased the granulation tissue region (>65%) and reduced the epithelial gap (>30%) in the full-thickness wound of the mice [103]. The healing mechanism could be the enhancement of the macrophage pro-healing function. In a human study evaluated by McDaniel et al. [104], the small blisters on the forearms were created to examine the effect of the daily intake of DHA (1.1 g) and EPA (1.6 g) on the healing rate. A significantly greater IL-1β expression was detected in the blister fluid of the DHA/EPA group than in the control. It is hypothesized that the increased proinflammatory cytokines at the wound site may be responsible for wound healing.

Since the appropriate inflammation in the wound area promotes the cell migration and skin tissue repair, the AA precursors, such as omega-6 and omega-9 fatty acids, may be responsible for the healing process because of their role as inflammatory modulators. Cardoso et al. [105] demonstrated that ALA (omega-3), LA (omega-6), and oleic acid (omega-9) modulated skin wound healing at different levels. The omega-9 fatty acid induced faster wound closure than omega-3 and omega-6 fatty acids. The wound treated with oleic acid followed by LA presented less edema compared with the control. Pereira et al. investigated the effect of LA and oleic acid on the inflammatory response of skin wounds and the cytokine release by rat neutrophils [106]. The animals treated with topical LA or oleic acid displayed a 60% greater reduction in the necrotic cell-layer thickness than the control. The number of neutrophils in the wound site was increased by LA (19.3) and oleic acid (24.6) as compared to the control (10.8). Oleic acid could stimulate the production of cytokine-induced neutrophil chemoattractant in inflammation 2 α/β (CINC-2α/β). Rodrigues et al. [107] investigated the effect of oral LA (0.22 g/kg) for improving wound healing in streptozotocin-induced diabetic rats. LA reduced the wound area 14 days post-induction. The increased CINC-2α/β, TNF-α, and leukotriene B_4_ and the increased leukocyte accumulation and angiogenesis by LA were responsible for the improved wound closure in the early healing phase. Laser ablation on the skin for an aesthetic regimen is often associated with erythema, edema, and crusting. Thirty-four subjects receiving fractional CO_2_ laser treatment were enrolled for topical application of conjugated LA to determine the healing efficacy [108]. Conjugated LA showing the ability to stimulate keratinocyte proliferation and epidermal regeneration was practical to reduce edema and itching at day 3 post-irradiation. The skin tolerated the topical conjugated LA well with no increased adverse effects. We summarize the information about the effect of fatty acids in fish oils on cutaneous wounds in Table 4.

### 3.5. Hyperpigmentation

Melanogenesis is a process of generating and distributing melanin, which is synthesized in melanocytes in specialized membrane-bound organelles known as melanosomes [109]. In the biosynthesis of melanin, tyrosinase is a rate-limiting enzyme catalyzing the conversion of tyrosine to 3,4-dihydroxyphenylalanine (DOPA) and the oxidization of DOPA to dopaquinone [110]. Skin hyperpigmentation by melanogenesis is stimulated by UV exposure, endothelin-1, α-melanocyte-stimulating hormone (α-MSH), growth factors, and cytokines [111]. Balcos et al. conducted a cell-based study [112] to explore the effect of DHA on melanin synthesis by using B16F10 melanoma as the cell model. DHA at 1–25 μM did not influence cell viability but decreased α-MSH-activated melanin production. Microphthalmia-associated transcription factor (MITF) is a predominant regulator for tyrosinase expression [113]. The results showed that DHA significantly increased tyrosinase degradation without affecting MITF expression.

ALA and LA are reported to reveal skin-whitening capability through the mechanism of tyrosinase inhibition [114]. Ando et al. [115] evaluated the impact of ALA and LA on hyperpigmentation suppression in the skin. Hyperpigmentation was induced by UVB (1 J/cm^2^) in guinea pigs. After a 3-week application, the lightness value (L*) of the skin was increased from 40.6 (UVB-treated control) to 47.1 and 48.8 by ALA and LA, respectively. The melanin content decreased to 16.4% and 28.0% compared with the control after ALA and LA treatment. Shigeta et al. [116] prepared LA-loaded liposomes as the carrier for skin whitening in humans. The hyperpigmentation of the volunteers was induced by UVB exposure (1.2x MED) on the forearm. The whitening effect was greater for liposomal LA (0.1%) than for free LA according to the measurement of L*. Liposomal encapsulation was applicable for the protection of unstable LA from oxidation.

## 4. Conclusions

Fish oil and the related actives, such as omega-3 and omega-6 PUFAs, have been proved helpful for maintaining skin homeostasis and ameliorating cutaneous abnormalities. The fatty acids in fish oil can improve skin barrier function, inhibit UV-induced inflammation and hyperpigmentation, attenuate dry skin and pruritus elicited by dermatitis, accelerate skin wound healing, and prevent skin cancer development. All the benefits can be achieved by different administration routes, including oral supplementation, topical application, and intravenous injection. Despite the evidence indicating the successful application of fish oil and omega-3 PUFAs on skin disorders, there have been conflicting reports from meta-analysis and systematic review regarding the clinical benefit of using fish oil over the control or other lipids. Fish oil is a crude extract with very complex ingredients. It is difficult to control fish oil contents well. The abundant sources of the fish genus also complicate the quality control. The specific fish type and the PUFA percentage in the fish oil are the important factors that should be considered for the benefits on the skin. Another issue that should be considered is that not only PUFAs but also vitamin A, vitamin D, retinol, selenium, and other components may contribute to the bioactivity of fish oil. The most commonly raised concern for omega-3 PUFA administration is the potential to raise the risk of bleeding via the anti-platelet effect. Gastrointestinal disturbance by dietary fish oil is also reported in some cases. Caution should be used in optimizing the benefits of fish oil or omega-3 fatty acids to ensure a balance between damage or toxicity and the effectiveness. Although many fish oil products and PUFAs are developed for testing in cell- and animal-based studies, clinical trials for skin application are still limited. This may be because of the high cost of clinical trials and some unknown side effects that should be identified and explored first. Further clinical studies are encouraged for future application of improved therapy.

## Figures and Tables

**Table 1:** The fatty acids existing in fish oils for attenuating cutaneous photoaging.

Composition	Experimental Model	UV Type	Benefit	Reference
DHA and EPA	Keratinocytes and skin fibroblasts	UV 270–400 nm, 25–100 mJ/cm^2^	Reduced IL-8	Storey et al. [43]
DHA	Keratinocytes	UV 290–400 nm, 10–60 mJ/cm^2^	Reverted cell resistance to UV-induced apoptosis	Serini et al. [44]
EPA	Skin fibroblasts	UV 275–380 nm, 25–75 mJ/cm^2^	Reduced MMP-1, ERK, and JNK	Kim et al. [46]
EPA	BALB/c mouse with ear edema	UVB, 500 mJ/cm^2^	Suppressed ear edema by oral EPA administration	Danno et al. [47]
DHA	Hairless mouse with skin inflammation	UVB 312 nm, 180 mJ/cm^2^	Decreased COX-2 and NOX-4 by blocking MSK1	Rahman et al. [48]
DHA	Hairless mouse with skin inflammation	UVB 312 nm, 180 mJ/cm^2^	Elevated Nrf2 activation and upregulation of cytoprotective genes	Yum et al. [50]
Fish oil rich of DHA and EPA	Human	UVB with a filter to eliminate wavelengths <295 nm	Increased minimal erythema dose and decreased serum triglyceride	Orengo et al. [51]
Fish oil rich in DHA and EPA	Human	UV 270–400 nm	Increased minimal erythema dose and decreased PGE2	Rhodes et al. [52]
Fish oils from mackerel, sardine, and horse mackerel	Human	Broadband UVB, doubled MED	Inhibited UVB-induced erythema	Puglia et al., [53]
Ethyl esters of EPA and oleic acid	Human	UV 270–400 nm	Increased minimal erythema dose and decreased PGE2	Shahbakhti et al. [54]
EPA	Human	UV 270–400 nm, 4 × MED	No function on Langerhans cell migration and PGD2 expression	Pilkington et al. [56]
EPA	Human	UV 285–350 nm	Decreased epidermal thickness, procollagen, COX-2, and MMPs	Kim et al. [57]
LA and ALA	Hairless mouse	UVB at 312.5 nm, 3.6 × MED	Lowered erythema score and PGE2 in skin	Takemura et al. [58]
Conjugated LA	Keratinocytes	UV 270–400 nm, 25–100 mJ/cm^2^	Reduced IL-8 and PGE2	Storey et al. [61]

COX-2, cyclooxygenase-2; ERK, extracellular-signal-regulated kinase; JNK, Jun-N-terminal kinase; LA, linoleic acid; MED, minimal erythema dose; MMP, metalloproteinases; MSK1, stress-activated kinase-1; NOX-4, nicotinamide adenine dinucleotide phosphate (NADPH): oxidase-4; PGD2, prostaglandin D2; PGE2, prostaglandin E2.

**Table 2:** The fatty acids existing in fish oils for preventing or treating cutaneous carcinogenesis.

Composition	Experimental Model	Tumor-Induced Approach	Benefit	Reference
DHA and EPA	Keratinocytes HaCaT	Growth factors in 3% FBS	Induced pre-malignant keratinocyte apoptosis	Nikolakopoulou et al. [66]
DHA	Melanoma A-375	Standard culture medium	Synergistic growth inhibition combined with NSAIDs	Chiu et al. [67]
Fish oil, DHA, and EPA	BCC TE 354 and SCC A431	Standard culture medium	Synergistic growth inhibition combined with imiquimod	Rehman et al. [70]
Fish oil	Swiss albino mouse	DMBA-induced papilloma	Reduced tumor size and enhanced IL-10	Rehman and Zulfakar [71]
Fish oil	Swiss albino mouse	Benzo(a)pyrene and croton oil	Reduced papilloma number per mouse	Ramesh and Das [74]
Fish oil	Hairless mouse	UV 280–320 nm, 30 mJ/cm^2^	Reduced size of papilloma, keratoacanthoma, and carcinoma	Lou et al. [75]
Omega-3 and omega-6 fatty acids	Human	SCC patients	Lower SCC risk with the higher omega-3/omega-6 intake	Hakim et al. [76]
Fish oil rich in omega-3 PUFAs	Human	Melanoma patients	Lower melanoma risk with the fish oil intake	Fortes et al. [77]
DNA-paclitaxel conjugate	Human	Melanoma patients	Prolonged median survival period	Homsi et al. [78]
Conjugated LA	Mouse	DMBA- and TPA-induced tumor	Reduced PPAR-δ and K-FABP	Belury et al. [81]

BCC, basal cell carcinoma; DMBA, 7,12-dimethylbenz[a]anthracene; FBS, fetal bovine serum; K-FABP, keratinocyte fatty acid binding protein; LA, linoleic acid; NSAIDs, non-steroidal anti-inflammatory drugs; PPAR-δ, peroxisome proliferator-activated receptor; SCC, squamous cell carcinoma; TPA, 12-O-tetradecanoylphorbol-13-acetate.

**Table 3:** The fatty acids existing in fish oils for preventing or treating dermatitis.

Composition	Experimental Model	Dermatitis-Induced Approach	Benefit	Reference
Fish oil	Rat	Acetone-induced dry skin	Increased skin hydration and scratching	Barcelos et al. [84]
Omega-3 PUFAs	Rat	Trimellitic anhydride-induced allergy	Decreased ear thickness, cutaneous eosinophils, and mast cells	Abdel Latif et al. [87]
DHA and AA	Mouse	Ovalbumin-induced dermatitis	Reduced Ki67 and elevated IL-10 expression	Weise et al. [88]
GLA	Human	Mild atopic dermatitis	Reduced TEWL and improved stratum corneum index	Kawamura et al. [91]
Dihomo-GLA	NC/Nga mouse	Dermatitis-like skin lesion	Suppressed clinical severity score and scratching behavior	Kawashima et al. [93]
Dihomo-GLA	NC/Tnd mouse	Dermatitis-like skin lesion	Upregulated PGD1 and reduced scratching behavior	Amagai et al. [94]

AA, arachidonic acid; GLA, γ-linolenic acid; PGD1, prostaglandin D1; PUFAs, polyunsaturated fatty acids; TEWL, transepidermal water loss.

**Table 4:** The fatty acids existing in fish oils for preventing or treating cutaneous wounds.

Composition	Experimental Model	Wound-Induced Approach	Benefit	Reference
Fish oil	Pig	Full-thickness skin excision	Fast wound closure at day 2	Shingel et al. [99]
Fish oil	Rat	Full-thickness skin excision	Accelerated healing process and increased IL-10	Peng et al. [101]
DHA	Rat	Full-thickness skin excision	Accelerated healing process and increased GPR120 and TGF-β	Arantes et al. [102]
14*R*,21-dihydroxy-DHA and 14*S*,21-dihydroxy-DHA	Mouse	Full-thickness skin excision	Increased granulation tissue region (>65%) and reduced epithelial gap	Lu et al. [103]
DHA and EPA	Human	Blisters in the forearms	Increased IL-1β expression in the wound sites	McDaniel et al. [104]
ALA, LA, and oleic acid	Mouse	Full-thickness skin excision	Faster wound closure by oleic acid than ALA and LA	Cardoso et al. [105]
LA and oleic acid	Rat	Full-thickness skin excision	Reduced necrotic cell layer thickness	Pereira et al. [106]
LA	Rat	Streptozotocin- induced diabetic wound	Increased leukocyte accumulation and angiogenesis	Rodrigues et al. [107]
Conjugated LA	Human	Fractional laser ablation	Reduced edema and itching	Wu and Goldman [108]

ALA, α-linolenic acid; GPR120, G-protein-coupled receptor 120; LA, linoleic acid; TGF-β, transforming growth factor β.
